# Computational analyses to reveal the key determinants of the high malignancy level of cholangiocarcinoma

**DOI:** 10.1515/jtim-2024-0033

**Published:** 2025-01-10

**Authors:** Xuan Li, Aoran Liu, Xuechen Mu, Zhihang Wang, Jun Xiao, Yinwei Qu, Zhenyu Huang, Ye Zhang, Ying Xu

**Affiliations:** Key Laboratory of Symbolic Computation and Knowledge Engineering of Ministry of Education, College of Computer Science and Technology, Jilin University, Changchun 130012, Jilin Province, China; The First Laboratory of Cancer Institute, The First Hospital of China Medical University, Shenyang 110001, Liaoning Province, China; College of Mathematics, Jilin University, Changchun 130012, Jilin Province, China; School of Medicine, Southern University of Science and Technology, Shenzhen 518055, Guangdong Province, China

**Keywords:** cholangiocarcinoma, bile acid, sialic acid, Fenton reaction, cancer migration

## Abstract

**Background and Objectives:**

Cholangiocarcinoma (CHOL) is a rare and highly aggressive cancer that originates in the bile duct; it has an average five-year survival rate of 9%, which makes it the cancer with the lowest survival rate among all 33 cancer types in the cancer genome atlas (TCGA) Program. The aim of this study is to elucidate the key determinants of the high malignancy level of CHOL through computational and cell-based experimental approaches and, particularly, to investigate how bile acids (BAs) influence CHOL’s propensity to metastasize.

**Methods:**

Our study analyzed the transcriptomic data from 1835 tissue samples of 7 digestive system cancer types in the TCGA database and compared them with those of 330 control tissue samples. Multiple cellular and molecular factors were considered in the study, including the level of hypoxia, level of immune cell infiltration, degree of cellular dedifferentiation, and level of sialic acid (SA) accumulation on the surface of cancer cells. Using these factors, we developed a multivariable regression model for the five-year survival rate, as reported by the Surveillance, Epidemiology, and End Results (SEER) Program reports, and analyzed how BA biology influences a few of these factors and causes CHOL to have a high malignancy level.

**Results:**

CHOL exhibited the highest level of SA accumulation and B-cell infiltration among all cancer types studied. BAs inhibit the cell cycle progression through the receptor *GPBAR1*, thereby limiting the rate of nucleotide biosynthesis—which in turn forces the cells to increase SA biosynthesis in order to maintain the intracellular pH at a stable level—thereby driving cell migration and metastasis, as established in our previous study.

**Conclusions:**

BAs are the key contributors to the lowest five-year survival rate of CHOL among the seven cancer types studied here. This finding not only reveals the molecular mechanisms underlying the high malignancy level of CHOL but also provides a new potential target for the diagnosis and treatment of CHOL.

## Introduction

Cholangiocarcinoma (CHOL) is a heterogeneous group of rare malignant cancers that occur in bile duct epithelial cells. It is the second largest subtype of liver cancer after hepatocellular carcinoma, accounting for approximately 15% of all primary liver cancers and 3% of all gastrointestinal cancers.^[1]^ In the past, this cancer has been misdiagnosed as liver cancer.^[2,3,4]^ The commonly used term “bile duct cancer” refers to all cancers that occur in the bile duct system. Due to its indolent, insidious, highly invasive, and drug-resistant characteristics,^[5]^ patients are typically diagnosed when the disease is already in an advanced stage, which limits the treatment options and leads to poor prognosis.^[5,6,7]^ Although researchers have made progress in understanding the biology, diagnosis, and treatment of CHOL, the prognosis of CHOL patients has not considerably improved in recent decades, with an average five-year survival rate of 9% and a high recurrence rate after tumor resection.^[5,8,9,10,11,12,13,14]^ Statistics on CHOL reveal that in 2017, there were 210,878 new cases and 173,974 deaths worldwide.^[15]^ While it has been well established that CHOL is highly malignant, the factors that lead to the high malignancy of CHOL remain largely unknown. Here we present a computational study of the transcriptomic data on the cancer with the aim of elucidating the main causes of the high malignancy of CHOL, which could potentially help with early diagnosis and improved treatment of the cancer.

To demonstrate that the cancer is highly malignant, we conduct a comparative study of CHOL *vs*. cancers in other digestive organs—that is, the colon, esophagus, liver, pancreas, rectum, and stomach. We did not consider the small intestine, as it rarely develops cancer.^[16]^ The scientific question we address is what makes CHOL the most malignant cancer compared to other cancers of the digestive system based on the five-year survival rates in Surveillance, Epidemiology, and End Results (SEER) reports (see Table 1).^[17,18]^ While pancreatic cancer (PAAD) has been considered the king of malignant cancer, CHOL has a lower five-year survival rate than PAAD.

**Table 1:** Five-year survival rates of seven cancer types of the digestive system^[17,18]^

Cancer type	Five-year survival rates			

	Localized	Regional	Distant	Combined
CHOL	23%	9%	3%	9%
COAD	91%	72%	13%	63%
ESCA	47%	26%	6%	21%
LIHC	36%	13%	3%	21%
PAAD	44%	15%	3%	12%
READ	90%	74%	17%	68%
STAD	72%	33%	6%	33%

Note: Localized, regional, distant, and combined refer to cancers that are entirely localized, have invaded nearby regions, are distantly metastasized, and all three types combined, respectively. CHOL: cholangiocarcinoma; COAD: colon cancer; ESCA: esophagus cancer; LIHC: liver cancer; PAAD: pancreatic cancer; READ: rectum cancer; STAD: stomach cancer.

Previous studies on possible contributors to the malignancy level of cancer include hypoxia,^[19,20]^ immune cell infiltration,^[21]^ and mutation burden.^[22]^ In this study, we have examined the following microenvironmental conditions in cancer tissues: (i) level of hypoxia; (ii) infiltration levels by neutrophils, macrophages, and B cells; (iii) level of dedifferentiation; and (iv) level of sialic acid (SA) accumulation on the cancer cell surface. The following is the rationale for considering these factors: (a) Hypoxia has long been known to be associated with poor prognosis for cancer.^[23]^ Hypoxia is generally the result of high levels of H_2_O_2_ and $\begin{array}{c} \cdot O_{2}^{-} \end{array}$ produced by local neutrophils and macrophages via consumption, which tends to be associated with more serious tissue damage and prolonged inflammation;^[24,25,26]^ (b) B cells in cancer tissues have been known to negatively regulate antitumor immunity and, hence, dampen antitumor responses.^[27,28]^ Therefore, a higher infiltration level of B cells tends to be associated with a poorer prognosis;^[29,30,31,32]^ (c) Dedifferentiation is a key mechanism utilized by differentiated cells for survival under persistent and extreme stressors by gaining more powerful capabilities that are available only to their less differentiated progenitor cells.^[33]^ Published studies have revealed that dedifferentiation has been widely used across different cancer types;^[34]^ and that (d) it was discovered in the 1960s that cancers with higher levels of SA biosynthesis tend to be more prone to metastasis,^[35,36]^ and later studies on SA *vs*. cancer metastasis have been predominantly focused on the signaling roles of SA.^[37,38,39]^

We have previously developed a model in which the increased level of SA biosynthesis gives rise to a higher level of SA accumulation on cancer cell surfaces,^[40,41]^ leading to increasingly stronger electrostatic repulsion among neighboring cancer cells as each SA carries a negative charge.^[42]^ Published studies have established that mechanical compression, as in cell-cell repulsion, can lead to morphological changes that activate the epithelialmesenchymal transition (EMT) program and lead to the migration of the affected cells,^[41]^ which is the first step in cancer metastasis.

We have estimated the level of each of the factors outlined in points (i) - (iv) based on the expression levels of the relevant marker genes for each cancer tissue across all seven cancer types and demonstrated that the five-year survival rate of each cancer type could be well represented as a function of the expression levels of these factors through a regression analysis, with high statistical significance. Key steps that lead to cell migration in CHOL are experimentally validated.

## Materials and methods

### Data

We have conducted comparative analyses of transcriptomic data of 1835 cancer tissue samples of 7 cancer types *vs*. those of 330 control tissues, all from the cancer genome atlas (TCGA) database *via* the genomic data commons (GDC) data portal (https://portal.gdc.cancer.gov).^[43]^ The detailed information of the datasets is given in Table 2.

**Table 2:** The numbers of the samples for the seven cancer types used in this study

Cancer type	#Tumor samples	#Control samples
CHOL	35	9
COAD	483	41
ESCA	185	13
LIHC	374	50
PAAD	179	4
READ	167	177
STAD	412	36

CHOL: cholangiocarcinoma; COAD: colon cancer; ESCA: esophagus cancer; LIHC: liver cancer; PAAD: pancreatic cancer; READ: rectum cancer; STAD: stomach cancer.

For each of the following factors analyzed, we have used the expression levels of the marker genes as widely in the literature (see Supplementary Tables S1 and S2): (a) the level of hypoxia;^[44,45]^ (b) the level of dedifferentiation;^[46]^ and (c) the levels of SA biosynthesis and degradation, nucleotide *de novo* biosynthesis, and Fenton reactions in each of the seven cancer types.^[46]^

### De-batch effect

We have applied an empirical Bayesian method to estimate the batch effect by modeling the relationship between gene expression levels and batch variables.^[47,48,49]^ The estimated batch effect is then subtracted from the raw data^[50]^ to ensure that transcriptomic data collected separately can be compared directly with each other.

### Single sample gene set enrichment analysis (ssGSEA) for pathway enrichment of individual samples

To perform gene set enrichment analysis (GSEA) on a single sample, we used the “gsva ()” function in R,^[51]^ with the analysis method set to “ssgsea”, which is specifically designed for GSEA on individual samples.^[52]^ The method is a non-parametric one that uses the empirical cumulative distribution function of the ranks of gene expressions within and outside a specified gene set to calculate the enrichment score. This method has been shown to be highly effective in identifying gene sets or pathways enriched by the given genes.^[53,54]^

### Estimation of SA accumulation on cancer cell surface

It is known that surface O-glycosylated proteins are involved in cancer metastasis.^[55,56]^ Hence, we have estimated the level of SA accumulation onto O-glycosylated proteins *via* sialyltransferases—that is, *ST6GALNAC1*, *ST6GALNAC2*, *ST6GALNAC3*, *ST6GALNAC4*, and *STA8SIA6*.^[57]^ It must be noted that only *ST6GALNAC1*, *ST6GALNAC2*, and *ST6GALNAC4* are upregulated in digestive cancers; hence, these are the only ones considered in our analyses. In addition, *NEU3* is the main SA degrader. We have used the following equation to estimate the average level of SA accumulation in stage-i samples for a target cancer type:

$$\begin{array}{c} \Delta SA_{i}^{\prime }=\frac{\sum_{G_{1}}Ave_{i}\left(G_{1},cancersamples\right)-Ave_{i}\left(G_{2},cancersamples\right)}{\sum_{G_{1}}Ave_{i}\left(G_{1},controlsamples\right)-Ave_{i}\left(G_{2},controlsamples\right)}, \end{array}$$ (1)

Where G_1_ = {*ST6GALNAC1*, *ST6GALNAC2*, *ST6GALNAC4*}, G_2_ = *NEU3*, and Ave_i_ (G, S) represents the expression of G averaged across all samples S in stage i. Further, the estimated level of SA accumulation up to stage I is expressed in the following manner:

$$\begin{array}{c} \Delta SA(I)=\sum_{i\leq I}\Delta SA_{i}^{\prime }, \end{array}$$ (2)

where I = 1 for localized tumor samples, 2 for regional tumor samples, and 3 for distant tumor samples, assuming that the duration of each of the three stages is approximately the same.

### xCell for estimating the infiltration levels by immune cells

xCell is a widely-used computational tool for calculating an enrichment score by the expressions of marker genes for each of the 64 immune and stroma cell types in a given cancer tissue sample.^[58]^ Using the calculated enrichment scores, we have estimated the infiltration levels of B cells, neutrophils, and macrophages in each sample by assessing each enrichment score against a total enrichment score for the tissue sample.

### Mechanisms framework

We have also considered bile acids (BAs) in our analysis. BAs can activate the G-protein-coupled BA receptor *GPBAR1*, which is known to suppress cell proliferation, particularly in cancer cells.^[59,60]^ This has an important implication in our analyses, which follow a specific chain of inference, described hereafter. (1) All cancer tissues in TCGA harbor the following persistent Fenton reaction:

$$\begin{array}{c} H_{2}O_{2}+\cdot O_{2}^{-}\;\overset{Fe^{2+}}{⟶}\;OH^{-}+\cdot OH+O_{2}. \end{array}$$ (3)

This reaction is harbored in the cell cytosols due to chronic inflammation (chemically, with elevated levels of H_2_O_2_ and $\begin{array}{c} \cdot O_{2}^{-} \end{array}$), coupled with local iron accumulation, which continuously produces OH^–^.^[61]^ (2) The continuous alkalization by the persistent Fenton reaction casts a life-threatening stress on the affected cells, thus driving the cells to start multiple acidifying reprogrammed metabolisms to keep the pH stable.^[61,62]^ (3) The two predominant reprogrammed metabolisms are the *de novo* biosynthesis of nucleotides (NTs) and biosynthesis and deployment of SAs by all cancers in TCGA, and together they have the following:

$$\begin{array}{c} R\left(OH^{-},FR\right)=R\left(H^{+},NT\right)+R\left(H^{+},SA\right)+\varepsilon , \end{array}$$ (4)

Where R (OH^–^, FR) represents the rate of OH^–^ production by Fenton reactions in cancer tissue, R (H^+^, NT) and R (H^+^, SA) are the rates of H^+^ production by nucleotide de novo biosynthesis and SA biosynthesis, respectively, and *ε* represents the total s produced by all other acidifying reprogrammed metabolisms, which is a relatively small quantity compared to R (H^+^, NT) + R (H^+^, SA).

A direct implication of the above equation is that if R (H^+^, NT) in cancer cannot be as high as that in other cancers for any reason, R (H^+^, SA) must be sufficiently high so their sum matches a major fraction of R (OH^–^, FR) to keep the pH stable. Our previous study has provided strong evidence that the increased SA biosynthesis and deployment drives cancer metastasis.^[41,63]^ The above analysis serves as the basis for our study in the results section.

### Principal component analysis (PCA) for geneexpression analyses

PCA is used to capture the main direction of change in a multi-marker gene set in terms of the changes in their collective expressions.^[64]^ Here, we use PCA analysis on the marker genes involved in the dedifferentiation process to quantify the level of dedifferentiation.

### Bayesian information criterion (BIC) for contribution estimation

The BIC is used to select the best model among the given options,^[65]^ it is defined in the following manner:

$$\begin{array}{c} BIC=k\times ln(n)-2ln(L), \end{array}$$ (5)

Where L is the maximum likelihood function of the model, *n* is the sample size, and k is the number of free parameters in the model.

The level of contribution to a model (model_K_) by the i^th^ free parameter is estimated using |BIC_K_|-|BIC_i_|, where BIC_K_ is the quality of the optimal model_K_ that is measured using the R^2^ score and BIC_i_ is the best model based on all parameters except for the i^th^ parameter. Hence, the percentage of contribution by the i^th^ parameter can be calculated in the following manner:

$$\begin{array}{c} Contribution_{i}=\frac{\left|BIC_{K}\right|-\left|BIC_{i}\right|}{\sum_{\left(i\leq K\right)}\left|BIC_{K}\right|-\left|BIC_{i}\right|} \end{array}$$ (6)

### Multiple linear regression (MLR)

MLR is used to model the linear relationship between a single dependent variable and multiple free variables. We used BIC-based MLR to perform a linear regression on the five-year survival rate against a list of possible contributors in each sample across the seven cancer types, where BIC provides information regarding the level of contribution to the regression result by each free variable.

### Cell culture

RBE and HuCC-T1 cells were obtained from Procell and cultured in RPMI-1640 medium supplemented with 10% fetal bovine serum (FBS, Moregate) and 1% pen-strep antibiotic (Procell). All cells were cultured in a humidified incubator at 37°C with 5% . First, we performed STR testing on the two cell lines to prevent misidentification or cross-contamination of cell lines. STR testing uses primers to extract repeated DNA fragments, usually two to six base pairs of tandem repeats. A DNA kit is used to extract high-purity DNA; thereafter, multiple rounds of PCR amplification are performed—that is, multiple gene loci are amplified. Finally, sequencing is performed to determine the experimental conditions.

### Generating persistent Fenton reactions in cytosol in cultured cells

FeSO_4_ (Sigma, F8633), H_2_O_2_ (Aladdin, 7722-84-1), and L-ascorbic acid (Sigma, A4544) were added to the culture to induce sustained Fenton reactions. Here we used L-ascorbic acid instead of $\begin{array}{c} \cdot O_{2}^{-} \end{array}$ as the reducing molecule for the persistent Fenton reaction.

### Cell proliferation and colony formation assays

Cell Counting Kit 8 (CCK-8, Apexbio) assays were performed to assess cell proliferation in accordance with the manufacturer’s instructions; 5000 cells were seeded in 96-well plates in triplicate containing regular fullserum media and allowed to adhere overnight. Different concentrations of lithocholic acid (LCD, MCE) were added the next day, and cells were harvested at the indicated times. Further, cells were incubated with 10% CCK-8 solution at 37°C for two hours, and then the absorbance was measured at 450 nm using a microplate reader.

For the colony formation assay, 600-800 cells were seeded in six-well plates in triplicate and adhered overnight. After 15 days, cells treated with LCD were fixed with polyformaldehyde (4%), washed twice with phosphate buffer saline (PBS), and stained with crystal violet solution (1%) for 15 min at room temperature.

### SA assay

The culture medium of cancer cells was collected as the acidic medium. SA was quantified using a Sialic Acid Assay kit (Abcam, ab83375) with the colorimetric assay protocol in accordance with the manufacturer’s instructions.

### Statistical analysis

Experiments were performed using a minimum of three replicates for each experimental group. For all statistical analyses, GraphPad Prism software (version 8.0) was used. Unpaired or paired Student *t* tests were used to compare data between the two groups. Statistical significance was defined as *P* < 0.05 (^*^P < 0.05; ^***^P < 0.001; ^****^P < 0.0001). All observations were expressed as mean SEM.

## Results

### CHOL is among the most hypoxic cancer types vs. controls

**Figure 1:**
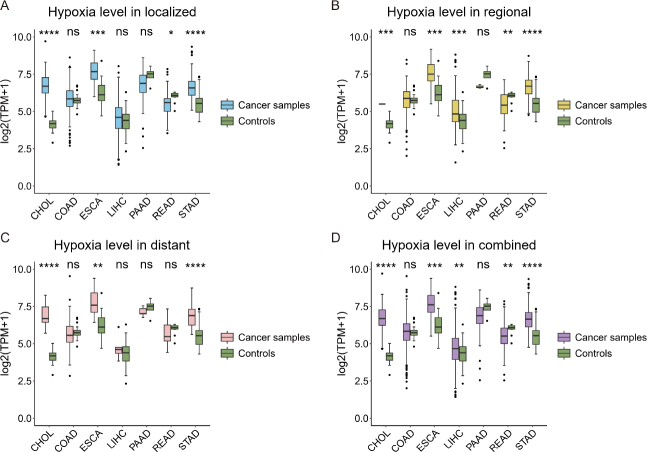
Hypoxic levels of cancer *vs*. control samples in localized (A), regionally migrated (B), distantly metastasized (C), and all cancer samples combined (D), where the expression of *HIF1A* is used to reflect the hypoxia level for cancer vs. control samples at each cancer stage across seven cancer types. The y-axis in each panel represents the level of log-transformed expression, measured using TPM. CHOL: cholangiocarcinoma; COAD: colon cancer; ESCA: esophagus cancer; LIHC: liver cancer; PAAD: pancreatic cancer; READ: rectum cancer; STAD: stomach cancer.

We used the expression level of *HIF1A* to reflect the level of hypoxia in a tissue sample, with a higher *HIF1A* expression indicating a higher level of hypoxia.^[45]^ Figure 1 depicts the expression levels of *HIF1A* in samples in localized, regionally migrated, distantly metastasized, combined cancer samples, and control samples for each of the seven cancer types, respectively. It is evident from the figure that CHOL has the largest increase in the expression of *HIF1A* in cancer *vs*. control samples across all stages, thereby indicating that CHOL is the most hypoxic cancer type among the seven.

### CHOL has the highest level of B-cell infiltration

**Figure 2:**
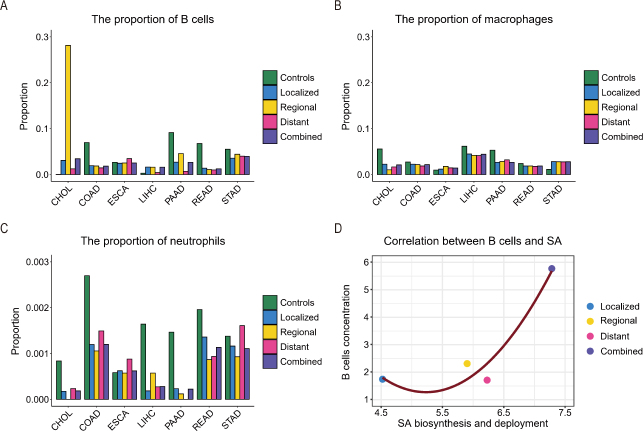
The estimated infiltration levels of (A) B cells, (B) macrophages, and (C) neutrophils in the control, localized tumors, tumors with regional migration, tumors with distant metastasis, or combined cancer samples for each of the seven cancer types, where the y-axis represents the percentage of the cells under study in a tissue sample. (D) The relationship between the infiltration level of B cells (the y-axis) and the level of SA biosynthesis and deployment (the x-axis), measured using the first principal component of the expressions of the marker genes of SA biosynthesis and deployment across the relevant samples, and the two have a correlation coefficient 0.823, given in Table S1. CHOL: cholangiocarcinoma; COAD: colon cancer; ESCA: esophagus cancer; LIHC: liver cancer; PAAD: pancreatic cancer; READ: rectum cancer; STAD: stomach cancer; SA: sialic acid.

We calculated the infiltration levels of B cells, macrophages, and neutrophils in each tissue sample using the xCell program (see materials and methods) in the control, localized, regionally migrated, distantly metastasized, and combined cancer samples for each of the seven cancer types. Figure 2 presents the calculation results, from which it is evident that CHOL has the highest level of B-cell infiltration *vs*. the controls across all seven cancer types on average.

To understand why CHOL has the highest level of B-cell concentration, a pathway enrichment analysis was undertaken to identify genes (Table S1, supplementary materials), whose expressions are highly correlated with genes involved in SA biosynthesis and deployment. Interestingly, B-cell activation is among the most correlated activities (Figure 2D). Therefore, we posit that the higher levels of SAs attract more B cells into the area, which is consistent with the results of published studies (SAs are the capping molecules of cell-surface glycans, which are part of gangliosides).^[66,67,68,69]^

### CHOL is among the most dedifferentiated cancers

**Figure 3:**
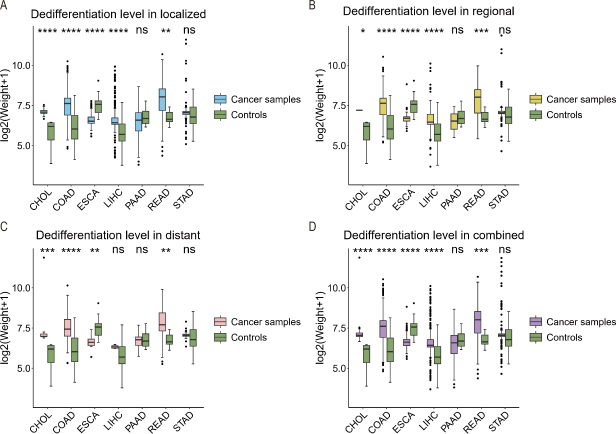
The level of dedifferentiation (the y-axis) is compared between cancer and control samples across the seven cancers in (A) totally localized tumor, (B) tumor with regional migration, (C) tumor with distant metastasis, and (D) combined cancer samples, where weight is the first principal component of the expressions of marker genes for dedifferentiation, where the gene list is given in Table S1. CHOL: cholangiocarcinoma; COAD: colon cancer; ESCA: esophagus cancer; LIHC: liver cancer; PAAD: pancreatic cancer; READ: rectum cancer; STAD: stomach cancer.

Using the marker genes for the level of cell dedifferentiation given in Table S1, we estimated the level of dedifferentiation *via* a PCA (see materials and methods). Figure 3 depicts the estimated level of cell dedifferentiation for each of the seven cancer types. It is evident from the figure that CHOL is among the cancer types that have the highest level of dedifferentiation, suggesting that the cancer is, in general, more stressed than other cancer types.

### CHOL has the highest level of SA accumulation on cancer cell surface

As discussed earlier, cancer cells utilize *de novo* biosynthesis of NTs and biosynthesis of SAs as the main H^+^ producers to neutralize OH^-^ produced by Fenton reactions.^[61,70]^

**Figure 4:**
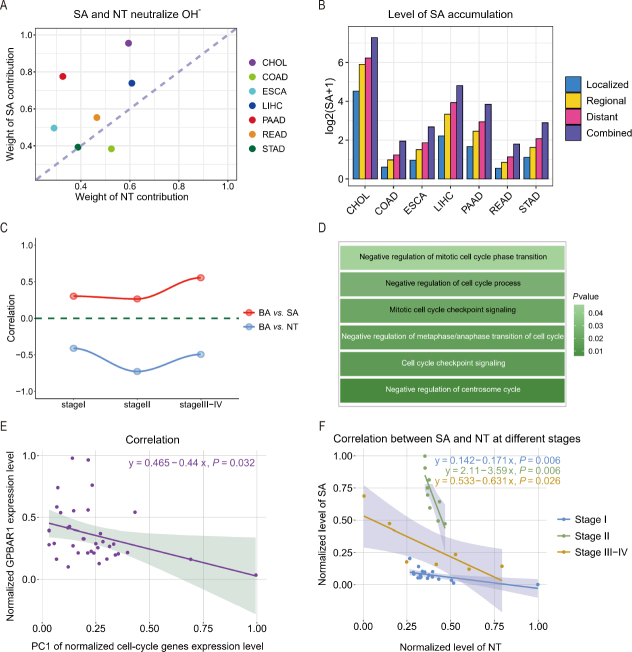
(A) Contributions by de novo nucleotide (NT) biosynthesis and SA biosynthesis and utilization in neutralizing OH^-^ produced by Fenton reactions, respectively, where the x-axis represents the weight of contribution by nucleotide biosynthesis and the y-axis represents the weight of contribution by SA biosynthesis and utilization, and a dot on the diagonal indicates that the two biosynthesis processes are equally weighted in the neutralization of the OH^-^ produced by Fenton reactions. (B) The predicted level of SA accumulation for each cancer type. (C) Correlations between BA biosynthesis and nucleotide de novo biosynthesis as well as between BA biosynthesis and SA biosynthesis. (D) Cell-cycle progression-related pathways enriched by genes that positively correlate with GPBAR1 in CHOL. (E) The BA’s receptor *GPBAR1* negatively correlates with cell-cycle marker genes, where each dot represents a CHOL sample with its normalized *GPBAR1* expression level (x-axis) and the normalized first principal component of the expression levels of cell-cycle genes (y-axis). (F) Correlation between the *de novo* nucleotide biosynthesis and the biosynthesis and utilization of SA in different stages. The x-axis represents the (normalized) level of *de novo* nucleotide biosynthesis, and the y-axis represents the (normalized) level of SA biosynthesis, where all the relevant marker genes are given in Table S1. CHOL: cholangiocarcinoma; COAD: colon cancer; ESCA: esophagus cancer; LIHC: liver cancer; PAAD: pancreatic cancer; READ: rectum cancer; STAD: stomach cancer; SA: sialic acid.

Figure 4A and 4B depicts the level of contribution of SA towards keeping the Fenton reaction-produced OH^–^ neutralized across the seven cancer types. We noted that CHOL stands out in terms of the level of contribution by SA biosynthesis. To understand why SA contributes particularly highly to keeping the cytosolic pH stable, we studied the roles that BAs may have played in this picture. First, we noted that (1) the level of BA, measured using the expression data of the relevant marker genes (Table S1), strongly correlates with the level of SA biosynthesis as well as the level of SA accumulation; and (2) the level of BA negatively correlates with nucleotide *de novo* biosynthesis, as depicted in Figure 4C, suggesting that BA might play a role in suppressing nucleotide synthesis and hence driving up the SA biosynthesis and deployment in accordance with equation (4).

To elucidate the possible connection between the two, we examined the expression level of the BA receptor *GPBAR1* and found that it is upregulated, which is consistent with the increased BA level in CHOL. Our literature search revealed that *GPBAR1* can suppress cell proliferation by activating the cAMP signaling pathway, which is known to be associated with cancer bone metastasis.^[71,72,73]^ To examine if this is the case in CHOL, we note that the expression of *GPBAR1* indeed negatively correlates with cell-cycle related pathways (Figure 4D, Table S1), and with the level of cell-cycle progression (Figure 4E), thereby confirming that *GPBAR1* suppresses cell proliferation in CHOL, as reported in the literature, which further limits the rate of nucleotide biosynthesis for DNA synthesis.

As noted earlier, nucleotide *de novo* biosynthesis and SA biosynthesis play the predominating roles in neutralizing the OH^-^s produced by Fenton reactions. The data depicted in Figure 4F explain why the SA biosynthesis level in CHOL stands out among the seven cancer types—that is, the nucleotide biosynthesis is suppressed as a result of suppressed cell proliferation by BAs *via* their binding with *GPBAR1*. Hence, it is the BA that ultimately causes the levels of SA biosynthesis and accumulation to go higher, thereby leading to the high migration rate by CHOL and, hence, the lowest survival rate among the seven cancer types. This computational prediction, the core of our model, is validated experimentally in the last section of results.

### Key factors contributing to the low survival rates of CHOL

We now aim to establish a quantitative relationship between the five-year survival rate and the possible contributing factors discussed earlier. To do this, we first predicted the possible causal relationships among these factors to identify and remove those factors that are largely determined by the other factors. Specifically, we sorted all the cancer samples of each cancer type in ascending order of the number of upregulated genes in a sample *vs*. controls, as a means to provide a pseudo-time order of the disease progression. On this ordered list of cancer samples, we used the F-test statistic in the Granger causality test^[74]^ to assess if some pairs are causally connected with a significant *P*-value. In other words, if the *P*-value of factor i to factor j is less than or equal to 0.05, we consider that factor i can predict factor j—that is, there is a causal relationship between the two. Table 3 lists the calculation results.

**Table 3:** Prediction of causal relationships between factors

Item	ΔSA	Hypoxia	Dedifferentiation	B cells	Macrophages	Neutrophils
ΔSA	1	0.059	0.5362	0.2756	0.0668	0.3459
Hypoxia	0.6139	1	0.7694	0.5222	0.9296	0.3864
Dedifferentiation	0.0001	0.0123	1	0.001	0.1245	0.1167
B cells	0.4243	0.0507	0.3472	1	0.0503	0.3736
Macrophages	0.6054	0.4275	0.0089	0.8036	1	0.132
Neutrophils	0.8353	0.5174	0.0757	0.5286	0.4279	1

It is evident from the table that the *P*-values of dedifferentiation relative to ΔSA, hypoxia, and B cells are all 0.05, indicating that there is a causal relationship between dedifferentiation and each of the three factors; hence, it was excluded from our regression analyses.

**Figure 5:**
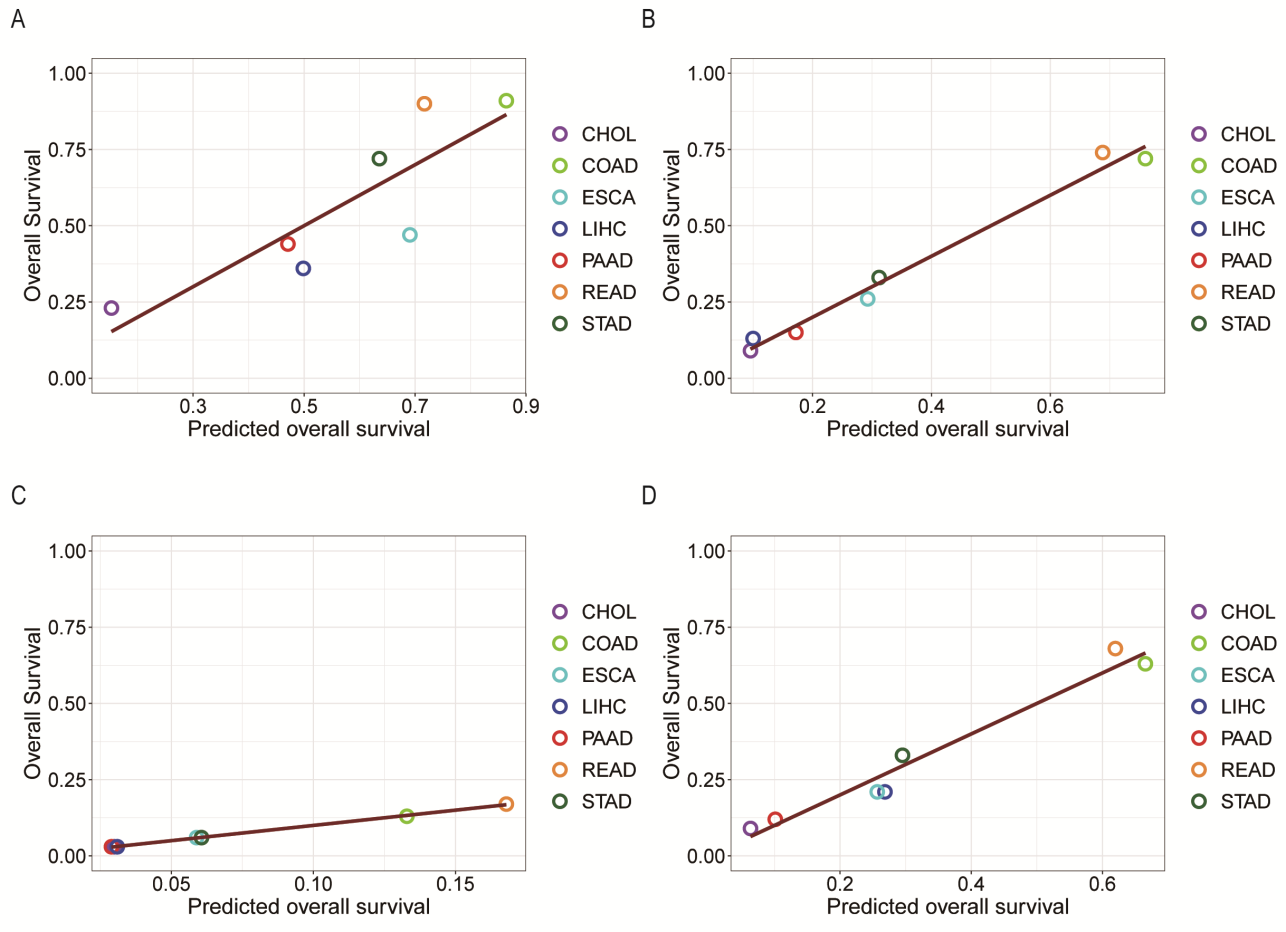
The five-year survival probabilities (y-axis) of seven types of cancers against the predicted survival probabilities (x-axis) based on SA accumulation, level of hypoxia, and the infiltration levels of B cells, macrophages, and neutrophils through regression analyses, with (A), (B), (C), and (D) representing localized tumors, tumors with regional migration, distant metastasis, and combined cancer sample set, respectively. CHOL: cholangiocarcinoma; COAD: colon cancer; ESCA: esophagus cancer; LIHC: liver cancer; PAAD: pancreatic cancer; READ: rectum cancer; STAD: stomach cancer.

We then conducted a linear regression analysis of the five-year survival rates using the five remaining factors: ΔSA; the level of hypoxia; the infiltration levels by B cell, neutrophil, and macrophage, respectively. Figure 5 presents the four regression models for localized (model_1_), regionally migrated (model_2_), distantly metastasized (model_3_) and combined cancer (model_4_) samples with the regression results, respectively.

$$\begin{array}{c} {\text{ Regression model }}_{1}:\text{SR}=1.0158-0.4043\Delta SA+0.2528H\\ +0.0434B+0.004M-0.4158N \end{array}$$ (7)

$$\begin{array}{c} {\text{Regression model}}_{2}:\text{SR}=1.3789-0.5405\Delta SA+0.3069H\\ +0.1469B-0.1133M-0.8068N \end{array}$$ (8)

$$\begin{array}{c} {\text{Regression model}}_{3}:\text{SR}=0.3679-0.1086\Delta SA-0.0948H+\\ 0.136B+0.0665M-0.124N \end{array}$$ (9)

$$\begin{array}{c} {\text{Regression model}}_{4}:SR=1.58-0.3908\Delta SA+0.0104H+\\ 0.2268B+0.0937M-0.6073N \end{array}$$ (10)

In the above equations, SR is the five-year survival rate; ΔSA is the levels of SA accumulation; H is the hypoxia; and B, M, and N are the infiltration level by B cells, macrophages, and neutrophils, respectively.

Our BIC-based analyses (see materials and methods) have provided the level of contribution by each of these factors, as given in Table 4. Hence, we conclude that the SA accumulation, dictated by the BA level and the level of Fenton reaction, plays the most significant role in making CHOL the deadliest cancer among the seven that were studied. Moreover, the infiltration level of B cells is the second most significant contributor to the lowest survival rate of CHOL, which is partially because of the high SA level, as discussed earlier.

**Table 4:** Performance results of regression models

Model	*R* ^2^	Contribution based on BIC analyses				

		ΔSA (%)	hypoxia (%)	B cell (%)	macrophage (%)	neutrophil (%)
model_1_	0.7287	65.1	14.59	3.51	1.09	15.71
model_2_	0.9843	42.86	7.81	35.87	0.27	13.19
model_3_	0.9991	49.03	5.21	37.8	0.53	7.43
model_4_	0.9628	52.06	0.17	26.48	4.33	16.96

### Experimental validation of Fenton reactions and cell-surface SA levels

The core logic of our predictive model is that it is the Fenton reaction that drives the high levels of nucleotide *de novo* biosynthesis and SA biosynthesis for survival and that it is the BAs in CHOL that suppress the level of cell proliferation, hence the level of nucleotide biosynthesis, which in turn drives the level of SA biosynthesis up to keep the intracellular pH stable for cells affected by Fenton reactions.

**Figure 6:**
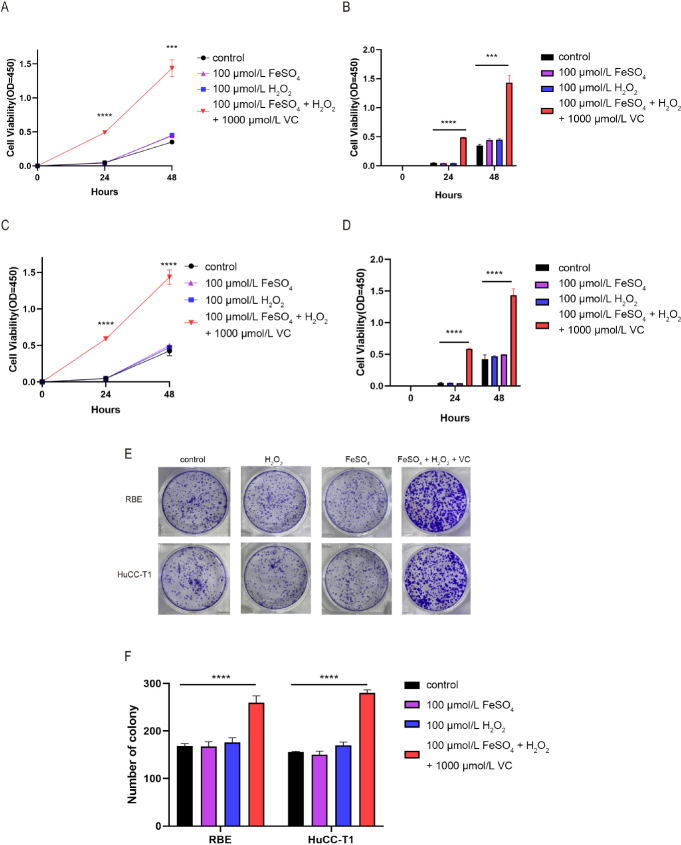
The sustained Fenton reaction promotes cell proliferation (Table S3). (A and B) The cell proliferation ability of the RBE cells was detected after adding FeSO_4′_, H_2_ O_2′_, and L-ascorbic acid. (C and D) The cell proliferation ability of the HuCC-T1 cells was detected after adding FeSO_4′_, H_2_ O_2′_, and L-ascorbic acid. (E) The colony formation assay was performed after adding, and L-ascorbic acid. (F) Statistical diagram of the colony formation assay (^****^P < 0.0001; ^***^P = 0.0002).

To verify this prediction, we selected CHOL cells (RBE and HuCC-T1 cells) for study. We first induced sustained cytosolic Fenton reactions in these cells by FeSO_4_, H_2_O_2_, and L-ascorbic acid (VC) to HuCC-T1, where L-ascorbic acid serves the same purpose of superoxide to reduce Fe^3+^ back to Fe^2+^, to induce persistent Fenton reactions. After preliminary experiments, we found that 100 μmol/L FeSO_4_, 100 μmol/L H_2_O_2_, and 1000 μmol/L L-ascorbic acid were the most suitable concentrations for inducing persistent Fenton reactions in cytosol. When FeSO_4_ was added in a concentration of 100 μmol/L, H_2_O_2_ in a concentration of 100 μmol/L, and L-ascorbic acid in a concentration of 1000 μmol/L, significantly increased cell proliferation was observed at 24 h and 48 h compared with the control group as result of increased nucleotide biosynthesis induced by Fenton reactions, and the difference was statistically significant (Figure 6 and Table S3).

**Figure 7:**
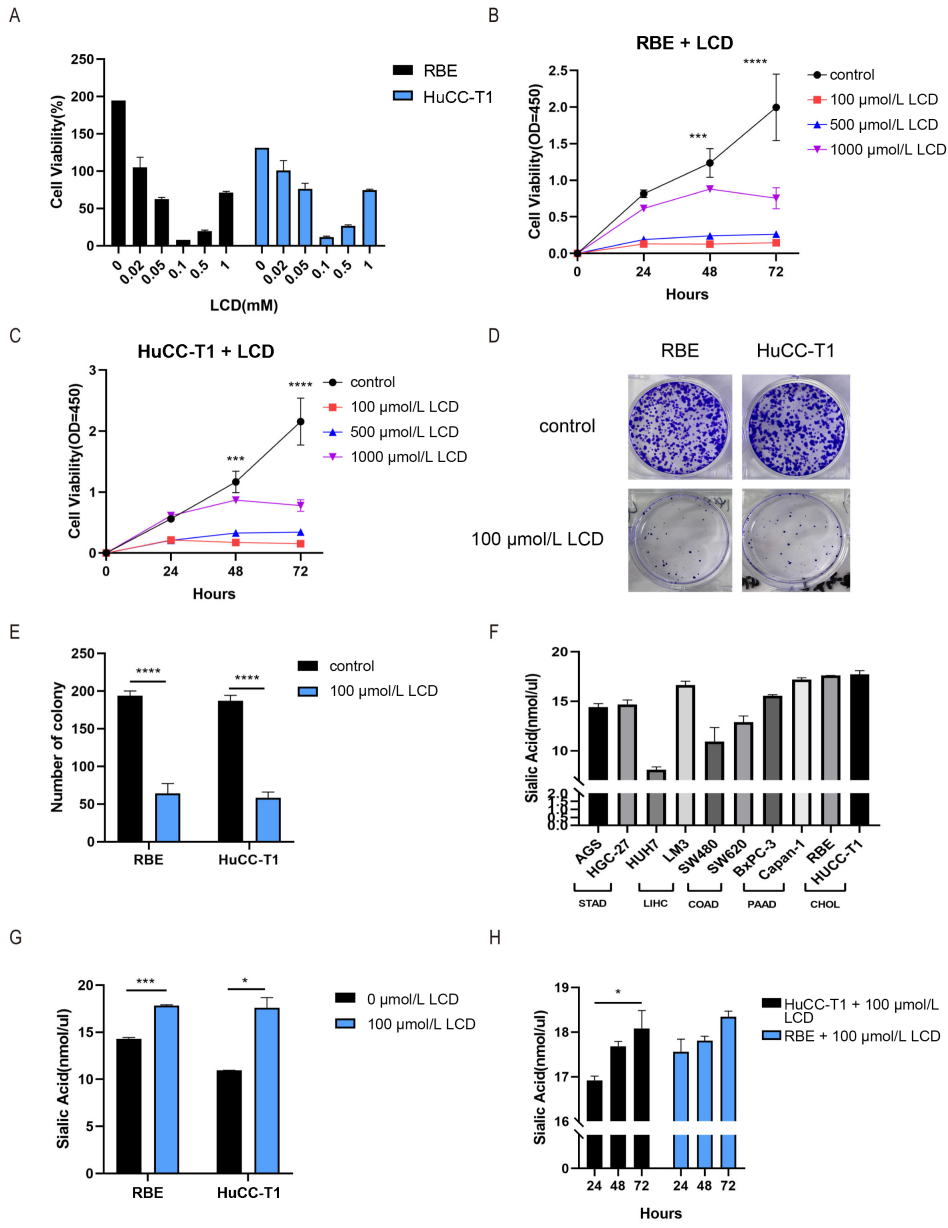
LCD inhibited proliferation of CHOL cells and increased cell-surface SA level (Table S4). (A) the cell viability (%) of CHOL cells was detected after adding different concentrations of LCD. (B and C) the cell proliferation ability of RBE cells and HuCC-T1 cells was detected after adding different concentrations of LCD. (D) the colony formation assay was performed after adding LCD. (E) statistical diagram of colony formation assay. (F) SA content (nmol/*μ*L) of different types of cancer cells. (G) the SA content (nmol/*μ*L) of CHOL cells after adding LCD was detected. (H) SA content (nmol/*μ*L) of CHOL cells at different time points after adding LCD. (^*^P < 0.05; ^***^P < 0.001; ^****^P < 0.0001.) SA: sialic acid.

We have then examined how the proliferating cells will be affected when treating them with the proliferation inhibitor LCD (Lithocholic acid) as in the case of BAs in CHOL. First, we note that the cell viability was the lowest when treated with the LCD with concentration at 100 μmol/L among other levels of concentrations (Figure 7A). Then, we tested the impact of different LCD concentrations on cell proliferation and found that LCD had the strongest inhibition of cell proliferation at the same concentration of 100 μmol/L for 48 h and 72 h (Figure 7B and 7C). Similarly, the colony formation assay found the same results (Figure 7D and 7E). Therefore, we chose the LCD concentration at 100 μmol/L for further experiments.

We then measured the SA level on the cell-surface in cells of stomach, liver, colon, and pancreatic cancer, together with CHOL cells, separately, and found that consistent with our above results, the highest SA content was found in CHOL cells (Figure 7F). Subsequently, we treated the CHOL cells with LCD at 100 μmol/L and observed that the cell-surface SA content continued to increase (Figure 7G) with time (*P*-value = 0.0174) (Figure 7H).

The conclusions are that (1) by inducing Fenton reactions in the cytosol of cancer cells, the cells proliferate at higher rates as predicted by our computational study and validated by our cell-based experimental study, and (2) by inhibiting experimentally the proliferation rate of such cancer cells, the cell-surface SA content increases as we have predicted, which drives cells to metastasize.^[41]^ This explains why CHOL is one of the most malignant cancers.

## Discussion

Different cancer types have intrinsically different malignancy levels such as pancreatic cancers are known to be the king of cancers and basal cell carcinoma generally does not kill people while other cancer types each tend to have a stable and distinct five-year survival rate across different patients. What determines the intrinsic malignancy level of a cancer type? An extensive literature review has revealed that very few studies have focused on such an important scientific question. Relevant papers tend to focus on specific microenvironmental factors such as the infiltration levels of innate immune cells or T cells, and the hypoxic level in a cancer tissue may be associated with the malignancy level within the same cancer type.^[75,76,77]^

We presented a comparative study among seven cancer types in the human digestive system to explain statistically why CHOL has the lowest five-year survival rate with high statistical significance compared to the six other cancer types. The information used is derived from the tissue-based transcriptomic data of a total of 1835 cancer tissue and 330 control tissue samples. Regression analyses coupled with Bayesian statistical analyses revealed that the top two contributors to the high malignancy level of CHOL are the high level of SA biosynthesis and deployment and the high level of B-cell infiltration. Interestingly the B-cell level is possibly due to the SA level, which is consistent with published literature.^[66,67,68,69,78,79]^ Hence, we conclude that it is the high SA level dictates the high malignancy level, which is consistent with published studies regarding the relationship between SA biosynthesis and cancer metastasis.^[41,80]^

Our in-depth analysis has revealed that the reason for the high SA level in CHOL tissues compared to the other digestive cancer types is due to the following. First, we have discovered that the key driving force for cancer development is the persistent alkalization of the intracellular pH due to continuous Fenton reactions,^[61,62,81,82]^ and the affected cells utilize predominantly nucleotide biosynthesis and SA biosynthesis to produce H^+^ to keep the pH stable, which is summarized in equation (4). Hence, we infer that it is the combination of these two processes that roughly balances the OH^-^ produced by Fenton reactions. Table 5 shows the levels of contributions by nucleotide biosynthesis and SA biosynthesis toward neutralizing the Fenton reaction level across the seven cancer types, using a BIC-based analysis. From equation (4) and the table, we conclude that (i) it is the lower contribution by nucleotide synthesis towards Fenton reaction in CHOL, compared to other six cancer types, that forces the affected cells to increase the contribution by SA biosynthesis as otherwise the cells will die from intracellular alkalosis; and (ii) it is the BA that represses cell cycle progression *via* the BA receptor *GPBAR1*, which negatively correlates with cancer cell-cycle progression revealed by our analyses and is known to repress the cell cycle,^[71,72,83]^ which limits the rate of nucleotide synthesis. This conclusion is strongly supported by our own experimental study and published studies.

**Table 5:** Contributions towards keeping the pH stable by SA and nucleotide synthesis

Cancer type	Contribution level by SA (%)	Contribution level by nucleotide synthesis (%)
CHOL	70.47	29.53
COAD	14.08	85.92
ESCA	69.12	30.88
LIHC	20.73	79.27
PAAD	70.09	29.91
READ	34.14	65.86
STAD	13.05	86.95

CHOL: cholangiocarcinoma; COAD: colon cancer; ESCA: esophagus cancer; LIHC: liver cancer; PAAD: pancreatic cancer; READ: rectum cancer; STAD: stomach cancer; SA: sialic acid.

Computational analysis revealed that our findings are consistent with previous studies in that hypoxia and immune cell infiltration are associated with cancer malignancy. In particular, we found that CHOL tissues had high levels of hypoxia, a known factor for poor prognosis in cancer, which is associated with increased tissue damage and chronic inflammation, which was most directly associated with *HIF1A* expression levels. In addition, we found that CHOL had the highest infiltration levels of B cells, which may be related to the increased levels of SA, as SA is a capping molecule of cell surface glycoproteins that attract B cells.

Dedifferentiation is a mechanism adopted by differentiated cells to survive under sustained and extreme stress by acquiring more powerful capabilities of their less differentiated progenitors, and our analysis suggests that CHOL is under great stress. However, dedifferentiation was excluded from the regression analysis with five-year survival because it was causally related to hypoxia, B cell infiltration, and SA accumulation levels.

Our study also found that CHOL cells had the highest levels of SA accumulation on the cell surface, which was associated with increased BA levels. BA plays a key role in inhibiting cell proliferation and indirectly driving SA biosynthesis and deployment by activating the *GPBAR1* receptor. This predicted causal inference relationship has been experimentally verified, that is, in the experimental verification, by inhibiting the proliferation of CHOL cells, the cell surface SA content increased.

Our study provides an in-depth understanding of the causes of CHOL as a highly malignant cancer, but there are still some limitations. Our study used transcriptomic data from the TCGA database, which may not accurately represent the activity levels of their encoded proteins. Experimental validation was mainly carried out *in vitro* cell models, which may not fully simulate the complex microenvironment in actual cancer tissues. Our future research will consider more omics data, and verify these findings in animal models, and investigate the possibility of inhibiting the function of *GPBAR1* as a drug target, which may reduce the level of malignancy of CHOL.

## Conclusion

The main contribution of this study is that it has demonstrated *via* an integrated computational and experimental study, for the first time, that it is the high level of SA biosynthesis and accumulation on the cancer cell surface, dictated by the BA level *via* its receptor *GPBAR1*, that makes the CHOL the deadliest cancer type among the seven cancer types studied. This is consistent with the general knowledge that higher BA levels tend to make liver cancer more malignant.

### Supplementary Information

Supplementary materials are only available at the official site of the journal (www.intern-med.com).

## Supplementary Material

Supplementary Material
