# Supplementary material for: Computational analyses to reveal the key determinants of the high malignancy level of cholangiocarcinoma
Source: J Transl Int Med. 2025 Jan 10;12(6):602–17. doi: 10.1515/jtim-2024-0033 (PMC12288948; doi:10.1515/jtim-2024-0033)
Supplement: Supplementary file 1 — Supplementary Material [file jtim-2024-0033_sm.pdf]

Supplementary table S1: Marker genes for interested microenvironment conditions	
**Cellular state or process**	**Marker genes**
Level of hypoxia	*HIF1A*
Dedifferentiation	*WNT3A, TRIM71, CTNNB1, GATA6, FOXO1, TBX5, SOX2, NOTCH3, MYCN, MYCL, STAT1, TWIST1, TWIST2, KLF5, KLF4, MYC, GDF11, SALL1, LIN28B, LIN28A, SALL4, PAX7, PAX6, POU5F1, STAT3, GATA4, SNAI1, NFIB, NOTUM, WNT1, ID2, NOTCH1, NOTCH2, ID1, MEF2C, STAT5A, MMP9, HES1*
SA transferase	*ST6GALNAC1, ST6GALNAC2, ST6GALNAC3, ST6GALNAC4, ST8SIA6*
SA degradation	*NEU3*
Level of BA	*ABCB11, GPBAR1, NR1C1, NR1C3, NR1D1, NR1I1, NR1I2, NR5A2, RXR, S1PR2, SLC22A1, SLC22A6, SLC22A7, SLC22A8, SLCO1B1, SLCO1B3, VDR*
Cell cycle	*CCNL1, CENATAC, KAT2A, CCNL2, CNTD1, ANAPC2, METTL3, CYP26B1, CCNB1IP1, NUF2, FKBP6, TAF1L, BUB1B, CDK1, PLK5, SPC25, E2F1*
B-cell activity level	*LEF1, EPHB2, CARD11, TNFRSF4, SYK, IL4I1, SWAP70, RASGRP1, PRKCD, LGALS1, BANK1, CD70, TGFB1, CMTM7, JAK3, TNIP2, TNFSF4, TNFSF13, TCIRG1, IL2RG, CTLA4, RNF8, CD19, SLAMF8, CD79A*
Cell proliferation genes in Stage I	*HDAC6, VIP, RREB1, GLUL, SIRT1, KDR, TEK, PROX1, PROK1, EGFR, APLNR*
Cell proliferation genes in Stage II	*SHH, HGF, DACH1, KDR, LIMS2, TEK, TIE1, KRIT1, EGR3, PROK2, MMRN2, MYC*
Cell proliferation genes in Stage III-IV	*IL10, STAT3, AKT1, SHH, TCF7L2, PIK3CD*
transferase genes in Stage I	*CDK1, TYMS, MELK, EXO1, AURKA, NEK2, DTYMK, NME1, MAP4K4, UBE2T, TK1, BUB1, CHEK1, RRM2, CKS1B, CDKN3, GMPS*
transferase genes in Stage II	*ALOX5, MGAT3, TRIO, CKB, ALDH3B2, XYLT1*
transferase genes in Stage III-IV	*FKBP10, PDE10A, TYRO3, HS3ST1, PGK1, BLVRA, PADI3, ADCY3*

**Supplementary table S2: Marker genes for processes of interested microenvironment conditions**			
**Cancer type**	**Marker genes**		
	**FR**	**SA**	**NT**
CHOL	*UBL7, SUMO2, IKBKB, TMUB2, ATG9B, RPS27A, RNPS1, FKBP1A, UBE2M, UBE2C, CDC20, UBE2S, UBL4A, SACS, UBFD1, NXT1, LSM2, ATG4B*	*NANS, ST6GALNAC4, ST6GALNAC2*	*ADA, CAD, CTPS2, NME5, UPP1, NME7, NME1, NME9*
COAD	*SUMO2, RNPS1, FKBP1A, UBE2C, UBE2S, UBL4A, UHRF1, UBFD1, NXT1, UBQLN4*	*NANP, ST3GAL2, ST8SIA2*	*HPRT1, PRTFDC1, APRT, PPAT, PRPS1, GART, SHMT2, GMPS, CAD, CMPK1, UMPS, UPP2, UCK2, NME6, NME1, NME2, DHODH*
ESCA	*UBL7, UBD, BECN1, FKBP1A, UBE2C, UBL4A, SACS, UHRF1, UBFD1, NXT1*	*ST3GAL2, ST6GALNAC4, ST8SIA4*	*PAICS, HPRT1, PRTFDC1, PRPS1, GART, GMPS, CAD, CTPS2, CMPK1, UCK2, NME2P1, DCK, NME7, NME9*
LIHC	*UBL7, SUMO2, ATG9B, RNPS1, FKBP1A, UBE2M, CDC20, UBE2S, UBL4A, SACS, NXT1, UBQLN4, LSM2*	*NANP, NANS, ST3GAL3, ST3GAL2, ST8SIA4*	*PRTFDC1, PRPS1, ADA, GMPS, CTPS2, UMPS, UCK2, DCK, UPP1, NME7, NME1, NME3*
PAAD	*SUMO2, UBD, BECN1, RNPS1, FKBP1A, UBE2S, UHRF1, NXT1, LSM2*	*ST6GAL2, ST6GAL1, NANP, ST3GAL1, ST6GALNAC3, ST6GALNAC1*	*PAICS, HPRT1, ADA, CTPS1, CMPK1, CDA, UMPS, UPP2, UCK2, NME4, UPP1, NME7, NME1*
READ	*SUMO2, UBD, BECN1, FKBP1A, UBE2C, UBE2S, UBL4A, UBFD1, NXT1, LSM2*	*ST6GAL2, GNENANP, ST3GAL2, ST8SIA2*	*HPRT1, APRT, PPAT, PRPS1, ADA, GART, SHMT2, CAD, CTPS2, CMPK1, UMPS, UCK2, NME6, NME1, DHODH*
STAD	*SUMO2, RNPS1, UBE2S, UBL4A, SACS, UHRF1, UBFD1, NXT1, LSM2*	*NANP, ST3GAL2, ST6GALNAC5, ST6GALNAC4*	*PAICS, HPRT1, PPAT, SHMT2, SHMT1, GMPS, CAD, CTPS1, UMPS, UCK2, NME6, NME2P1, DCK, NME1*

Supplementary table S3: The sustained Fenton reaction promotes cell proliferation												
**Fenton reaction-RBE-CCK8<br>The cell proliferation ability of the RBE cells was detected after adding FESO4, H2O2, and L-ascorbic acid**												
Hours	control			100uM H2O2			100uM FESO4			100uMFESO4+100uMH2O2+1000uMVC		
0	0	0	0	0	0	0	0	0	0	0	0	0
24	0.0452	0.0443	0.0547	0.0452	0.0421	0.0437	0.0457	0.0422	0.0447	0.4865	0.4848	0.4953
48	0.3572	0.3304	0.3678	0.4376	0.4397	0.4734	0.4252	0.4685	0.4382	1.5535	1.4453	1.3059
**Fenton reaction-HUCCT1-CCK8<br>The cell proliferation ability of the HuCC-T1 cells was detected after adding FESO4, H2O2, and L-ascorbic acid**												
Hours	control			100uM H2O2			100uM FESO4			100uMFESO4+100uMH2O2+1000uMVC		
0	0	0	0	0	0	0	0	0	0	0	0	0
24	0.0432	0.0423	0.0577	0.0484	0.0429	0.0477	0.0437	0.0441	0.0449	0.5868	0.5814	0.5957
48	0.4572	0.3484	0.4671	0.4776	0.4729	0.4634	0.4953	0.4981	0.4972	1.3531	1.5457	1.4056
**Fenton-colony<br>The colony formation assay was performed after adding FESO4, H2O2 and L-ascorbic acid**												
	control			100uM H2O2			100uM FESO4			100uMFESO4+100uMH2O2+1000uMVC		
RBE	163	173	169	172	155	174	187	168	172	253	276	249
HuCC-T1	156	154	157	158	149	143	169	177	163	273	281	286

Supplementary table S4: LCD inhibited proliferation of cholangiocarcinoma cells and increased cell-surface SA level

The cell viability (%) of cholangiocarcinoma cells was detected after adding different concentrations of LCD

mM	RBE			HUCC-T1		
0	0.9467	0.912	0.9726	0.5277	0.582	0.6209
0.02	1.0847	1.0715	1.1108	0.6419	0.6529	0.7418
0.05	0.9834	0.9339	0.8947	0.5873	0.6834	0.7213
0.1	0.1659	0.1651	0.1736	0.211	0.1852	0.1789
0.5	0.1685	0.1773	0.225	0.23	0.2073	0.1874
1	0.5993	0.6033	0.6438	0.5752	0.6289	0.6353

The cell proliferation ability of RBE cells is detected after adding different concentrations of LCD

Hours	control			100umLCD			500uMLCD			1000uMLCD		
0	0	0	0	0	0	0	0	0	0	0	0	0
24	0.769	0.803	0.8724	0.1271	0.1113	0.1526	0.1685	0.1773	0.225	0.5993	0.6033	0.6438
48	1.033	1.2532	1.4245	0.1282	0.1272	0.1319	0.2208	0.2484	0.2538	0.8568	0.8906	0.8931
72	1.4892	2.1402	2.3611	0.1361	0.152	0.1513	0.2535	0.2657	0.2731	0.5912	0.8081	0.8645

The cell proliferation ability of HuCC-T1 cells is detected after adding different concentrations of LCD

Hours	control			100umLCD			500uMLCD			1000uMLCD		
0	0	0	0	0	0	0	0	0	0	0	0	0
24	0.5749	0.5666	0.5426	0.2153	0.2068	0.2184	0.23	0.2073	0.1874	0.5752	0.6289	0.6353
48	1.3262	1.1982	0.9767	0.1801	0.1687	0.1692	0.3483	0.311	0.3271	0.8841	0.8518	0.8699
72	2.437	2.3138	1.7161	0.1526	0.1573	0.1497	0.3859	0.3415	0.3068	0.8485	0.8197	0.6693

SA content (nmol/ul) of different types of cancer cells

AGS	HGC-27	HUH7	LM3	SW480	SW620	BxPC-3	Capan-1	RBE	HUCC-T1
14.1694	14.362	7.85	16.91	11.924	12.431	15.469	17.323	17.58	17.9765
14.654	14.983	8.2649	16.379	9.9304	13.324	15.63	17.067	17.605	17.4578

The SA content (nmol/ul) of cholangiocarcinoma cells after adding LCD is detected

	0uM LCD		100uM LCD	
RBE	14.21823	14.40057	17.76095	17.87796
HUCC-T1	10.94975	10.95334	18.36755	16.85571

SA content (nmol/ul) of cholangiocarcinoma cells at different time points after adding LCD

Hours	RBE+100uM LCD		HuCC-T1+100uM LCD	
24	17.76095	17.35822	16.9878	16.85571
48	17.74013	17.87796	17.76095	17.60589
72	18.43503	18.25341	17.79899	18.36755

The colony formation assay is performed after adding LCD

	control			100uM LCD		
RBE	200	187	194	50	68	75
HuCC-T1	193	189	179	67	55	53
